# Editorial: Neurological mechanisms of empathy for distress

**DOI:** 10.3389/fpsyt.2025.1686354

**Published:** 2025-09-10

**Authors:** Qing Zhao, Shanquan Chen, Jing Meng

**Affiliations:** ^1^ State Key Laboratory of Cognitive Science and Mental Health, Institute of Psychology, Chinese Academy of Sciences, Beijing, China; ^2^ Department of Psychology, University of Chinese Academy of Sciences, Beijing, China; ^3^ School of Public Health, University of Hong Kong, Hong Kong, Hong Kong SAR, China; ^4^ Research Center for Brain and Cognitive Science, Chongqing Normal University, Chongqing, China; ^5^ Key Laboratory of Applied Psychology, Chongqing Normal University, Chongqing, China

**Keywords:** empathy for distress, empathy for pain, empathy for emotions, spiritual empathy, neurological mechanisms

Neurological Mechanisms of Empathy for Distress (July 2022–July 2025) was a special project proposed to investigate the neurological mechanisms underlying empathy for distress. Empathy is the sharing and understanding of others’ feelings (1, 2). It is a multidimensional concept, consisting of empathy for others’ physical sensations (e.g., empathy for pain), inner feelings (e.g., empathy for emotions), and spiritual thoughts (e.g., empathy for music) (3, 4). With a collaborative effort by 27 authors, in tandem with 17 reviewers and editors, this project was finalized, featuring six articles that illuminate the nature of empathy for distress.

As introduced in the following, the topics covered by these articles went broader than the initial design of the project. However, intriguingly, the theme of these studies converged with the concept of multidimensional empathy:

## Physical empathy

Li et al. studied the event-related potentials (ERPs) of empathy for pain, based on the Empathy for Pain Stimuli System (EPSS; 1). They observed that empathy for pain was modulated both by participant traits (e.g., autistic traits) and stimulus traits (e.g., static vs. dynamic stimuli). Essentially, they verified that the ERP components of empathy for pain were two-fold: early emotional components (N1, P2, and N2, which arise above the frontoparietal lobe) and late cognitive components (P3 and LPP, which occur above the parieto-occipital lobe). The former could represent an automatic emotional contagion of others’ pain, and the latter might represent a cognitive evaluation of the pain per se, as well as its potential consequences.

## Emotional empathy

Arioli et al. investigated the neurostructural bases of empathy for emotions. By focusing on grey matter volume through univariate and multivariate brain morphometry, they observed robust neural correlates of emotional empathy, but not for cognitive empathy. Moreover, distinct brain structures were identified between self-oriented (i.e., personal distress) and other-oriented emotional empathy (i.e., empathic concern). Personal distress involved the insula and amygdala, which might underpin one’s tendency to experience an automatic resonance with others’ distress, as well as inclination to withdraw from it for self-protection. Instead, empathic concern was associated with the medial precuneus and inferior parietal cortex, likely reflecting one’s ability to shift attention from oneself to others, thereby enabling prosocial behavior.

Lavin et al. studied the impact of the presence of a “third individual” on the prosocial reactions of young adults using the electroencephalogram (EEG) technique. They observed that when presented with a “third person” who is in need (i.e., a child potentially benefiting from the donation), participants were more likely to accept altruistic yet unfair monetary distribution. Using EEG, Lavin et al. further identified that the presence of the “third person” enhanced participants’ frontal theta oscillations. They pondered that the increased oscillations might be due to an inclusive empathic concern for others in general. In other words, being concerned about one individual may have a Ripple Effect, triggering empathy concerns for others’ well-being in a more general context.

## Spiritual empathy

Obando Yar et al. conducted a systematic review to explore the neurological basis of individuals’ perception of fictional characters. They reported that the neural activation related to the fictional resonance was qualified by the role of the characters (i.e., protagonists vs. antagonists): the appreciation of protagonists was followed by a stronger synchronization among empathy-related brain regions, such as the medial prefrontal cortex (mPFC) and the inferior frontal gyrus (IFG). In contrast, engaging with antagonists may lead to enhanced activation of brain regions involved in subjective value integration and comparison, including the anterior cingulate cortex (ACC). Overall, their findings indicated that moral resonance might serve as an amplifier of spiritual empathy.

## Cross-dimensional empathy

Pan et al. studied the impacts of death attitude and inadequate personal traits (e.g., antisocial and narcissistic personalities) on clinical empathy with trainee nurses. Clinical empathy stresses spiritual resonance with patients’ physical, emotional, and mental suffering due to illness, clinical treatments, and approaching death. They found that nurses with more serious personality inadequacies reported a lower level of clinical empathy. Furthermore, via mediation and moderation analyses, they found that these nurses’ adverse attitudes to death (i.e., fear, avoidance, and unrealistic beliefs) could underpin the above impairment. Ergo, Pan et al. suggested that maintaining a neutral and realistic attitude towards life and death is essential for promoting empathy among clinical professionals.

Vickers et al. innovatively studied a primeval–humane empathy towards charities. They presented participants with brief written introductions to charities and asked about their decisions on monetary donations. Behavioral results revealed that the donation was biased towards neonates with immediate nurturant needs over other forms of aid for adults. Neural imaging results further indicated that the above donation bias was relevant to motor-related brain regions (e.g., primary and supplementary motor areas). Vickers et al. deemed that care for neonates—closely related to species survival—predominantly are physical activities (e.g., feeding and cuddling). Hence, the above neurological evidence-based altruism bias towards neonates might have been prioritized by primitive communities, encoded in our brains, and passed on to our modern societies.

## For the future

Empathy is a multidimensional unity (3, 4). It ranges from the primary type of empathy for others’ externalized physical feelings (e.g., pain), to inner feelings (e.g., emotions), and ultimately to the innermost thoughts embodied in the forms of art, music, literature, and similar media (e.g., artistic expressions). The associations among these empathy unity elements, especially empathy for distress in the physical, emotional, and spiritual domains, warrant in-depth investigation. An understanding of their associations and potentially overlapped neural bases may eventually reveal the secrets underpinning the overall concept of empathy. Remarkably, with the development of artificial intelligence (5, 6), the above spectrum of empathy is anticipated to be expanded into AI-mediated empathy.
